# Development and validation of a health education e-book on scoliosis for adolescents

**DOI:** 10.1590/1984-0462/2026/44/2025352

**Published:** 2026-10-19

**Authors:** Beatriz dos Santos Oliveira, Ana Paula Oliveira Carnevalli de Miranda, Sílvia Maria Amado João, Patrícia Jundi Penha

**Affiliations:** a Pontifícia Universidade Católica de São Paulo, São Paulo, SP, Brasil.; b Universidade de São Paulo, São Paulo, SP, Brasil.

**Keywords:** Scoliosis, Adolescent, Health education, Teaching materials, Validation study, Escoliose, Adolescente, Educação em saúde, Materiais de ensino, Estudo de validação

## Abstract

**Objective:**

To describe the development and validation of a health education e-book for adolescents on adolescent idiopathic scoliosis.

**Methods:**

A methodological study divided into three phases: bibliographic survey and interviews for content analysis; development; and validation of the e-book by 36 adolescents (18 in each group: with and without adolescent idiopathic scoliosis). The e-book was validated using a questionnaire assessing four domains: comprehension and language, structure and illustration, reading motivation, and relevance. Descriptive statistical analysis was performed, and an agreement rate of 85% or higher was considered indicative of adequate e-book evaluation.

**Results:**

The e-book “Adolescent idiopathic scoliosis: What is it and how to treat it?” is digital, has 20 pages in the A5 half-page format, and was organized into topics. It is available for free. The domains assessed and the agreement percentages across the groups with and without adolescent idiopathic scoliosis, respectively, were text and language comprehension (92.1% and 100%), structure and illustrations (100% and 98.1%), motivation for reading (100% and 88.9%), and relevance (96.3% and 100%). Agreement was higher than 85% for all the domains assessed, showing a positive assessment.

**Conclusions:**

The health education e-book for adolescents on adolescent idiopathic scoliosis was considered valid.

## RESUMO

**Objetivo:**

Descrever a elaboração e validação de um *e-book* destinado a adolescentes para a educação em saúde sobre a escoliose idiopática do adolescente (EIA).

**Métodos:**

Estudo metodológico dividido em três fases: levantamento bibliográfico e entrevistas para análise de conteúdo, elaboração e validação do *e-book* por 36 adolescentes (18 em cada grupo: com e sem EIA). A validação foi realizada por meio de questionário que avaliou quatro domínios: compreensão e linguagem, estrutura e ilustração, motivação de leitura e relevância. Utilizou-se análise estatística descritiva, e a taxa de concordância, igual ou maior que 85%, indicou adequada avaliação do e-book.

**Resultados:**

O e-book “Escoliose idiopática do adolescente: o que é e como tratar?” possui 20 páginas, formato A5, conteúdo organizado em tópicos, com acesso digital e gratuito. Os domínios avaliados e os percentuais de concordância para os grupos com EIA e sem EIA, respectivamente, foram: compreensão de texto e linguagem (92,1% e 100%), estrutura e ilustrações (100% e 98,1%), motivação para leitura (100% e 88,9%) e relevância (96,3% e 100%). Em todos os domínios avaliados, atingiu-se um percentual maior que 85% de concordância, indicando avaliação positiva do material.

**Conclusões:**

O e-book desenvolvido foi considerado válido para ser usado na educação em saúde sobre EIA para adolescentes.

## INTRODUCTION

Adolescent idiopathic scoliosis (AIS) is a three-dimensional deformity of the spine, with a Cobb angle greater than or equal to 10º.^1^ This type of scoliosis has a multifactorial etiology, based on genetic, hormonal, biomechanical, and neurological theories.^2^ AIS affects adolescents from the age of 10, during the period of growth and skeletal maturation.^3^

Worldwide, the prevalence of AIS ranges from 2 to 3%.^3^ In the Brazilian context, the prevalence of AIS has been estimated at 1.5% in cities in the state of São Paulo, and 3.1% in the region of Pernambuco.^4^ ^,^ ^5^ A condition is classified as a public health problem based on three criteria: magnitude, transcendence, and vulnerability. Although AIS has low magnitude in terms of prevalence, it is characterized by high transcendence due to impairments, such as functional limitation, pain, and psychosocial factors caused by curve progression.^3^ ^,^ ^6^ ^,^ ^7^ ^,^ ^8^ ^,^ ^9^ In terms of vulnerability, the early detection and conservative treatment of AIS can prevent the progression of the scoliotic curve and the need for more invasive interventions.^3^ ^,^ ^6^ In Brazil, early identification of scoliosis remains a challenge, as school screening is not adopted as a public health policy.^4^ ^,^ ^5^

Therefore, health education about AIS is important for raising public awareness, promoting early diagnosis and appropriate treatment. Educational materials represent a viable alternative for transmitting knowledge to the population, facilitating the understanding of a more technical and scientific subject.^10^ ^,^ ^11^ ^,^ ^12^ ^,^ ^13^ ^,^ ^14^ ^,^ ^15^ ^,^ ^16^

However, few materials address AIS, and no science-based educational resource on AIS validated for the adolescent target population has been identified. Although some educational materials exist, none of them address topics described in the literature, such as the prevalence and complications of AIS, back pain, and mental health.^17^ ^,^ ^18^ ^,^ ^19^ The objective of this study was to describe the process of developing and validating an educational material, in e-book format, to promote health education about AIS for Brazilian adolescents.

## METHOD

This methodological study addressed the development of an educational material in e-book format aimed at adolescents and was organized into three phases: bibliographic survey and content analysis (phase 1), e-book development (phase 2), and validation by representatives of the target audience (phase 3).^16^ ^,^ ^20^ ^,^ ^21^

This study was approved by the Ethics Committee of the Pontifical Catholic University of São Paulo (CAAE: 69456423.9.0000.5482). All participants and their guardians read and signed the Informed Consent Form and the Understanding Form.

In phase 1, two researchers (one undergraduate physiotherapy student and one physiotherapist who is also a doctoral student) conducted a bibliographic survey on AIS to extract information for the e-book. The databases used were MEDLINE, SciELO, and LILACS. The keywords used were *escoliose, adolescente, colete, exercícios específicos para escoliose, and rastreamento* (and their English equivalents: scoliosis, adolescent, brace, specific exercises approach to scoliosis, and screening). Based on the results, the information and content selected for the e-book included: definition of the condition, prevalence, diagnosis, natural history, back pain, available treatments, sports practice, mental health, and guidance on whom to consult in case of suspected AIS.

A co-participatory development method was adopted, beginning with qualitative interviews to inform the e-book’s content analysis.^22^ The sample size for the interviews was based on qualitative research recommendations, which suggest three to six participants as sufficient for this type of study.^23^ The participants, interviewed via video calls (Google Meet), comprised 13 individuals divided into three groups: five adolescent girls with AIS undergoing conservative treatment (mean age 14.4±1.9), their mothers (mean age 44.2±6.1), and a multidisciplinary team of three experts (mean age 42.7±6.8): a physical therapist specialized in conservative treatment, a pediatric orthopedist, and an orthopedic surgeon specialized in scoliosis surgery. All volunteers were recruited through a convenience sample.

The inclusion criteria were:

1. Adolescents aged 10 to 17 years diagnosed with AIS and undergoing conservative treatment for at least one month;
2. Their mothers, participating as the person responsible for the adolescent’s diagnosis and treatment, such as accompanying them to medical appointments; and
3. Specialists, having at least two years of experience in treating adolescents with AIS.

The exclusion criteria were: unable to participate in video calls and/or read and evaluate the content of the e-book.

The responses obtained during the interview were recorded by the researcher in a Google Form and subjected to qualitative and quantitative analysis that included the calculation of the frequency of each response within the total sample.

Initially, all volunteers were asked: ‘What are the main challenges and difficulties of the AIS conservative treatment?’ Seven (54%) reported brace use - describing challenges such as discomfort, embarrassment, bullying, and difficulty with clothing; six (46%) mentioned adherence to and persistence with long-term treatment; and three (23%) pointed to the lack of access to quality information, limited access to care, and the high cost of AIS conservative treatment. In response to the question, ‘What information and guidance do you believe are important for an adolescent with scoliosis and their families?’ Five (38%) mentioned information on the indication and dosage of scoliosis treatment; four (31%) mentioned information on physiotherapeutic scoliosis-specific exercises and physical activity; and three (23%) mentioned information on early diagnosis and general information about scoliosis.

For phase 2, the e-book development team consisted of one undergraduate physiotherapy student and three physical therapists with experience in AIS research and treatment:

1. A physical therapist with a Master’s degree enrolled in a doctoral program, certified in the Scientific Exercises Approach to Scoliosis (SEAS), with eight years of research and clinical experience in AIS treatment;
2. A physical therapist with a doctoral degree, certified in the SEAS, with 15 years of research and clinical experience in AIS treatment; and
3. A physical therapist with a doctoral degree, and 24 years of research experience, 15 of which focused on AIS.

Based on the bibliographic survey and the data collected during the interview, the content to be included in the e-book was selected, and the topics were those described in phase one.

The health communication principles that guided the development of the e-book included ensuring content relevance, using simple, accessible language for the target audience, organizing the material in a logical sequence, and selecting illustrations and colors that aligned with the topic addressed.^20^ ^,^ ^21^ ^,^ ^24^ To support these principles, the Canva graphic design platform was chosen because it is free, offers a wide range of tools and illustration libraries, and is easy for the authors to use. The e-book’s color palette consisted mainly of shades of green, in reference to Scoliosis Awareness Month (Green June). The selection of illustrations, along with the layout and wording of the text, was intended to make the e-book visually appealing and appropriate for the target audience. The e-book was drafted by an undergraduate physiotherapy student and a physical therapist enrolled in a doctoral program, and reviewed by the two physical therapists with greater research experience in AIS.

The e-book was registered with ISBN number 978-65-83454-21-8 and is freely available, though copyrighted, only in Portuguese at https://colunafeliz.com.br/pdfs/E-BOOK-EIA.pdf.

For phase 3, the sample size of the study was determined using the infinite population sample calculation formula,^25^ given by n=Zα^2^×P×(1-P)/d^2^, because the adolescent target population for this validation - that is, the population likely to use the e-book - is large and cannot be fully enumerated. The 85% agreement threshold was established based on the literature on educational material validation. This is considered the minimum acceptable agreement value among evaluators of health education e-books.^24^ Considering a minimum proportion of 85% agreement on the relevance of each item evaluated and a 15% margin of difference in that agreement, the final calculation resulted in n=(1.96)^2^×(0.85)×(0.15)/(0.15)^2^, yielding a sample of 22 adolescents.^24^ However, adding 20% to account for sample loss, the final sample size was determined to be 28 adolescents, comprising 14 adolescents without AIS and 14 adolescents with a medical diagnosis of AIS. At the end of the validation phase, the study sample reached 36 adolescents - 18 with AIS and 18 without an AIS diagnosis. The invitations to recruit adolescent volunteers were sent on social media, such as Instagram, academic groups, and the Brazilian Scoliosis Society via WhatsApp.

The inclusion criteria for phase three (validation) were: age between 10 and 17 years, consistent with the chronological classification of AIS^3^, both sexes, and the ability to read and understand the e-book. Adolescents with AIS were required to have a medical diagnosis of AIS, whereas those in the group without AIS were required to have no prior diagnosis of scoliosis. The exclusion criteria were being illiterate or having any condition that impairs reading or understanding the e-book.

The Google Forms platform was used to create the validation questionnaire, which assesses four domains of the e-book: text comprehension and language, structure and illustrations, reading motivation, and relevance. Each domain consisted of three questions answered on a dichotomous scale: “yes - I agree” or “no - I disagree”. For a positive evaluation of all questions, agreement is considered ideal, except for question 1.2, in which disagreement is regarded as positive. At the end of each domain, a space was provided for suggestions and comments.^10^ ^,^ ^26^

In addition to the quantitative assessment of agreement, the open-ended responses provided by the adolescents were examined using content analysis. The comments were read independently by two researchers, who categorized the material according to the domains of the validation instrument.

Within the same platform, a link to the Google Drive application was included in the questionnaire. This link would direct respondents to the PDF version of the e-book for validation. The questionnaire responses were automatically programmed to populate a Google spreadsheet.

Descriptive statistical analysis was performed to characterize the sample and to summarize the adolescents’ responses to the validation questionnaire. Categorical variables were expressed as absolute and relative frequencies, and continuous variables (e.g., age) were described using means and standard deviations.

Given that the questionnaire items were measured on a dichotomous scale (yes/no), the primary analysis focused on the proportion of agreement for each item and each e-book domain. For question 1.2, disagreement was considered a positive evaluation, as previously defined.

For each domain (text comprehension and language, structure and illustrations, reading motivation, and relevance), the percentage of agreement was calculated based on the adolescents’ responses. Domain-level agreement percentages were calculated as the average of the agreement rates for each item within the domain, as seen in the Results section tables. An agreement rate of 85% or higher was considered indicative of adequate e-book validation, according to predefined criteria.

Comparisons between adolescents with and without AIS were conducted in an exploratory manner, based on the agreement proportions across groups, without formal hypothesis testing. This approach was adopted due to the small sample size and the categorical nature of the data, prioritizing an interpretative analysis aligned with the study’s validation purpose.

All analyses were performed using Stata version 13.0.

## RESULTS

The e-book has 20 pages, 15 of which are dedicated to AIS content. The pages are in A5 portrait format, measuring 14.8 cm wide x 21 cm high. The e-book cover is titled “Adolescent Idiopathic Scoliosis: What is it and how to treat it?” The table of contents allows adolescents to understand the topics covered in the material, facilitating reading. The text content is distributed in text boxes to make reading dynamic. The e-book’s color palette consists of shades of green to honor Green June (Scoliosis Awareness Month), and the illustrations are colorful and detailed (Figure 1)^27^.

**Figure 1.**
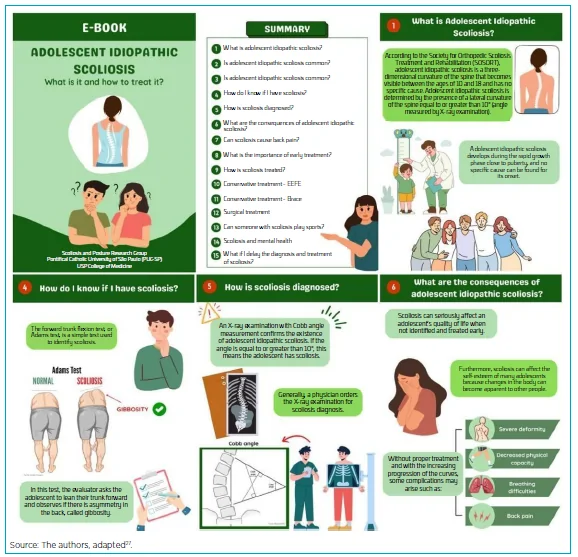
Pages from the e-book “Adolescent Idiopathic Scoliosis: What it is and how to treat it?”

Table 1 presents data on the characterization of the study sample. Table 2 presents the evaluation made by adolescents from both groups regarding the questionnaire domains. For both groups, agreement in all domains was acceptable, being above the 85% agreement threshold, and the groups showed similarities in their evaluation.

**Table 1.** Characterization of the sample of adolescents participating in the validation phase (phase 3) (n=36).

Characteristics	Group without AIS (n=18)	Group with AIS (n=18)
Age (years; mean ± SD)	15.5±2.0	13.6±2.0
Female sex [n (%)]	6 (33.3)	17 (94.4)
Occupation [n (%)]		
Student [n (%)]	18 (100)	18 (100)

AIS: adolescent idiopathic scoliosis.

Source: The authors.

**Table 2.** Evaluation of the e-book domains by adolescents with and without Adolescent Idiopathic Scoliosis (n=36).

Domains and questions*	Group with AIS (n=18) (%)	Group without AIS (n=18) (%)
Text comprehension and language	92.6	100
1.1. Were you able to understand the information about adolescent idiopathic scoliosis presented in this e-book?	18 (100)	18 (100)
1.2. Are there many technical words and words that are difficult to understand?	15 (83.3)	18 (100)
1.3. Can any literate adolescent understand the information presented?	17 (94.4)	18 (100)
Structure and illustrations	100	98.1
2.1. Are the text and illustrations well organized?	18 (100)	18 (100)
2.2. Do the illustrations help in understanding the information?	18 (100)	18 (100)
2.3. Does the e-book cover pique your interest in reading it?	18 (100)	17 (94.4)
Motivation for reading	100	88.9
3.1. Did you feel motivated to read the entire e-book?	18 (100)	15 (83.3)
3.2. Does the e-book contain information that will make adolescents want to learn more about adolescent idiopathic scoliosis?	18 (100)	15 (83.3)
3.3. Does the e-book motivate adolescents to prevent adolescent idiopathic scoliosis?	18 (100)	18 (100)
Relevance	96.3	100
4.1. Does the e-book provide sufficient information for adolescents about the prevention and diagnosis of adolescent idiopathic scoliosis?	18 (100)	18 (100)
4.2. Does the e-book offer sufficient information about the complications and treatments available for adolescent idiopathic scoliosis?	16 (88.8)	18 (100)
4.3. Do you consider the e-book relevant in helping to raise adolescent idiopathic scoliosis awareness among adolescents?	18 (100)	18 (100)

AIS: adolescent idiopathic scoliosis.

*All responses reflect ‘yes’ answers as positive evaluations of the e-book, except for question 1.2, for which the ‘no’ answer is considered a positive response.

Source: The authors.

Although all domains achieved an agreement rate above 85%, the comments provided during the validation phase were analyzed and, when applicable, incorporated. Table 3 lists the comments made by the adolescents who participated in the study, their corresponding domains, and whether the modifications suggested were implemented.

**Table 3.** Adolescents’ comments and suggestions analyzed for e-book modification.

Domain (D)	Comments and suggestions	Modification
Adolescents without AIS		
D2	“None, the layout is good, but the text could be a little smaller and with a font that makes reading easier”.	Accepted
D3	“It’s not that the e-book itself didn’t motivate me to read it entirely or that it doesn’t have information that makes adolescents want to learn more about adolescent idiopathic scoliosis, because it has all of that; it’s just that this subject is somewhat uninteresting to most young people these days. But with plenty of illustrations and eye-catching content, the e-book does grab my attention, making me want to read it, but as the e-book progresses, it gets a bit tiring”.	Not accepted
D3	“You could include practical examples and real-life cases to make the reading more interesting”.	Not accepted
D4	“Using the consequences of not treating the problem to attract more adolescents to treatment”.	Accepted
Adolescents with AIS		
D1	“Initially gibbosity and Cobb angle, but after reading it becomes understandable”.	Not accepted
D1	“Idiopathic”.	Not accepted
D1	“Measurement, idiopathic”.	Accepted
D2	“More details and more striking images of scoliosis itself”.	Accepted
D4	“To better explain the dangers and risks of not treating”.	Accepted
D4	“I believe that the complications of this problem could worsen if there is no treatment”.	Accepted

AIS: adolescent idiopathic scoliosis; D1: Text comprehension and language; D2: Structure and illustrations; D3: Reading motivation; D4: Relevance.

Source: The authors.

## DISCUSSION

This study described the development and validation of the e-book entitled “Adolescent Idiopathic Scoliosis: What it is and how to treat it?”, based on a co-participatory methodology that included adolescents with and without AIS. The inclusion of both groups aimed to explore whether prior familiarity with the condition could influence perception, comprehension, and relevance of the educational e-book.

Considering the descriptive nature of the validation process and the dichotomous format of the questionnaire, the analysis focused on the proportion of agreement across items and domains. Comparisons between groups were interpreted descriptively to identify differences in perception without performing inferential statistical testing.

During e-book development, the bibliographic survey and interview data were fundamental for selecting relevant AIS information and topics. These information-gathering strategies used to compose the health education material were also applied in other studies focused on the development of educational resources.^11^ ^,^ ^13^

The e-book wording employed understandable language for the adolescent audience. A simple and fluent writing style is essential when drafting educational health materials - not only to ensure comprehension but also to enhance persuasion and produce the desired effects. The e-book’s illustrations are eye-catching, contribute to the understanding of the topics addressed, and help maintain the reader’s attention and interest. The e-book has 20 pages. Compared with other studies in the literature, this material is about five to ten pages shorter.^10^ ^,^ ^12^ ^,^ ^13^ ^,^ ^14^

Including adolescents without AIS in the validation process was essential to ensure that the e-book was understandable and relevant not only to those living with the condition but also to adolescents with no prior knowledge of scoliosis. Adolescents with AIS may have greater familiarity with clinical terminology and treatment concepts, whereas adolescents without AIS may represent the first-contact perspective. This distinction allows a broader evaluation of the educational e-book in terms of clarity and accessibility for the adolescent population.

The mean age of the group without AIS was 15.5±2.0 years, and that of the group with AIS was 13.6±2.0 years. Regarding sex, the group without AIS consisted of 12 boys (66.7%) and six girls (33.3%), while the group with AIS included 17 girls (94.4%) and only one boy (5.6%). The higher prevalence among females and treatments targeting individuals who are skeletally less mature and therefore younger can explain these discrepancies.^4^ Although ideally the groups would have been matched by age and sex, the observed discrepancies between groups reflect the epidemiological profile of AIS. Despite these differences, both groups achieved agreement rates above the 85% threshold across all domains, suggesting that the e-book was well understood and positively evaluated regardless of age and gender.

Unlike some studies that relied on professional graphic designers, the e-book was designed by the research author and collaborators using the graphic design platform Canva, as it is free and easy to use.^10^ ^,^ ^11^ ^,^ ^13^ ^,^ ^14^ ^,^ ^15^ Despite not being designed by a specialized professional, the e-book validation results showed that for the domain of structure and illustrations, the findings exceeded the established threshold of 85% agreement in both groups of adolescents, reaching 98.1% (group without AIS) and 100% (group with AIS). Both the text and the illustrations were considered well organized and helpful in understanding the information. However, the cover of the material did not attract the attention of one adolescent without AIS (question 2.3), as occurred in another validation study of an educational booklet for the prevention of metabolic syndrome in adolescents, which also reported minor disagreement from part of the target audience regarding the cover design.^15^

For adolescents without AIS, the domain of reading motivation showed lower agreement (88.9%), as some adolescents did not feel motivated to read the entire e-book or did not have the desire to learn about AIS, resulting in 83.3% agreement for questions 3.1 and 3.2. In contrast, the group with AIS showed 100% agreement in reading motivation. The results of this study are comparable to those of another booklet drafting and validation study for adolescents, which addressed sustainable practices for promoting adolescent health, in which approximately 10% of the adolescents did not feel motivated to read the booklet until the end.^13^

Regarding the relevance domain, adolescents without AIS showed 100% agreement. As for adolescents with AIS, in question 4.2, two participants disagreed that the e-book provided sufficient information about the complications and risks if scoliosis were not properly treated, leading to 88.8% agreement for this question and 96.3% agreement for this domain. This perspective can be explained by the fact that adolescents diagnosed with scoliosis tend to have greater understanding and exposure to information about the condition. This creates a need for more detailed content. However, the purpose of this study was to develop educational material to raise awareness about scoliosis among adolescents in general.

Despite a positive evaluation in all domains, the adolescents’ suggestions and comments helped improve the final version of the e-book. This action promotes the inclusion of the target audience in critical reasoning about their health status, promoting the acquisition of knowledge and healthy behaviors.^13^ ^,^ ^14^ ^,^ ^15^

One study limitation is that the e-book was not clinically validated by a panel of expert judges on AIS, as observed in other studies.^10^ ^,^ ^12^ ^,^ ^13^ ^,^ ^14^ Although experts participated in phase 1 of the study, contributing to content analysis, they did not take part in its validation (phase 3). In the future, studies are expected to assess the effectiveness of the e-book in improving adolescents’ AIS knowledge.

Therefore, this e-book on AIS can be made available to disseminate knowledge and foster health-promoting actions among adolescents.

## Data Availability

The database that originated the article is available with the corresponding author.
